# Occult Pathologic Findings in Reduction Mammaplasty in 5781 Patients—An International Multicenter Study

**DOI:** 10.3390/jcm9072223

**Published:** 2020-07-13

**Authors:** Britta Kuehlmann, Florian D. Vogl, Tomas Kempny, Gabriel Djedovic, Georg M. Huemer, Philipp Hüttinger, Ines E. Tinhofer, Nina Hüttinger, Lars Steinstraesser, Stefan Riml, Matthias Waldner, Clark Andrew Bonham, Thilo L. Schenck, Gottfried Wechselberger, Werner Haslik, Horst Koch, Patrick Mandal, Matthias Rab, Norbert Pallua, Lukas Prantl, Lorenz Larcher

**Affiliations:** 1 Division of Plastic and Reconstructive Surgery, Department of Surgery, Stanford University, Stanford, CA 94305, USA; bkuehl@stanford.edu (B.K.); cbonham@stanford.edu (C.A.B.); 2 University Center for Plastic, Reconstructive, Aesthetic and Hand Surgery, University Hospital Regensburg and Caritas Hospital St. Josef, 93053 Regensburg, Germany; lukas.prantl@klinik.uni-regensburg.de; 3 Breast Health Center, General Hospital Merano, SABES South Tyrol, 39012 Meran, Italy; florianvogl@yahoo.com; 4 Division of Plastic and Reconstructive Surgery, Department of Surgery, Klinikum Wels-Grieskirchen, 4600 Wels-Grieskirchen, Austria; tomas.kempny@gmail.com; 5 Department of Plastic, Reconstructive and Aesthetic Surgery, Innsbruck Medical University, 6020 Innsbruck, Austria; gabriel.djedovic@lkhf.at; 6 Section of Plastic Surgery, Kepler University Hospital, 4020 Linz, Austria; ghuemer@drhuemer.com; 7 Department of Plastic, Reconstructive and Aesthetic Surgery, University Hospital St. Poelten, 3100 St. Poelten, Austria; philipp@huettinger.at; 8 Department of Plastic, Aesthetic and Reconstructive Surgery, Wilhelminen Hospital, 1160 Vienna, Austria; Ines.Tinhofer@khgh.at; 9 Department of Plastic and Reconstructive Surgery, Medical Institution Rudolfstiftung, 1030 Vienna, Austria; nina@huettinger.at; 10 Department of Plastic, Reconstructive and Aesthetic Surgery, European Medical School at the Carl von Ossietzky University of Oldenburg, Evangelisches Krankenhaus, 26122 Oldenburg, Germany; lars@steinstraesser.de; 11 Academic Teaching Hospital Feldkirch, 6800 Feldkirch, Austria; praxis@dr-riml.com; 12 Department of Plastic Surgery and Hand Surgery, University Hospital Zurich, 8091 Zurich, Switzerland; Matthias.Waldner@usz.ch; 13 Department for Hand, Plastic and Aesthetic Surgery, Ludwig–Maximilians University Munich, 80336 Munich, Germany; thilo.schenck@hotmail.de; 14 Department of Plastic, Aesthetic and Reconstructive Surgery, Hospital of the Barmherzige Brueder, Paracelsus Medical University Salzburg, 5020 Salzburg, Austria; g.wechselberger@me.com; 15 Division of Plastic and Reconstructive Surgery, Medical University Vienna, 1090 Vienna, Austria; office@haslik.at; 16 Clinic for Plastic, Aesthetic and Reconstructive Surgery, Department of Surgery, 8036 Graz, Austria; drkoch@plastische-op.at; 17 Klinikum Nuernberg, Department of Plastic-, Reconstructive-, and Hand Surgery, Center for Burn Injuries, Paracelsus Medical University, 90471 Nuremberg, Germany; dr.patrick.mandal@gmail.com; 18 Klinikum Klagenfurt am Woerthersee, Department of Plastic, Aesthetic and Reconstructive Surgery, 9020 Klagenfurt am Woerthersee, Austria; ordination@rab-plast.at; 19 Department of Plastic Surgery, Hand Surgery, Burn Center, University Hospital Aachen, 52074 Aachen, Germany; info@pallua.de; 20 Unit of Plastic Surgery, SABES, South Tyrol, 39042 Brixen, Italy

**Keywords:** reduction mammaplasty, occult breast cancer findings, international multicenter study, breast cancer screening

## Abstract

Breast cancer is among the most commonly diagnosed cancers in the world, affecting one in eight women in their lifetimes. The disease places a substantial burden on healthcare systems in developed countries and often requires surgical correction. In spite of this, much of the breast cancer pathophysiology remains unknown, allowing for the cancer to develop to later stages prior to detection. Many women undergo reduction mammaplasties (RM) to adjust breast size, with over 500,000 operations being performed annually. Tissue samples from such procedures have drawn interest recently, with studies attempting to garner a better understanding of breast cancer’s development. A number of samples have revealed nascent cancer developments that were previously undetected and unexpected. Investigating these so-called “occult” findings of cancer in otherwise healthy patients may provide further insight regarding risk factors and countermeasures. Here, we detail occult findings of cancer in reduction mammaplasty samples provided from a cohort of over 5000 patients from 16 different institutions in Europe. Although the majority of our resected breast tissue specimens were benign, our findings indicate that there is a continued need for histopathological examination. As a result, our study suggests that preoperative imaging should be routinely performed in patients scheduled for RM, especially those with risk factors of breast cancer, to identify and enable a primary oncologic approach.

## 1. Introduction

Breast cancer is the most commonly diagnosed cancer in women, afflicting 1 in 8 women during their lifetime [1], and the leading cause of cancer death among women [2]. Even in developing countries, the incidence of breast cancer is rapidly rising due to increasing life expectancy and dietary habits, among other risk factors [3]. Due to limited breast cancer awareness, most breast cancer are not detected at an early stage when treatment is likely to be more successful [4].

In 2018, the incidence rate for breast cancer was over two million worldwide [2]. To date, effective screening for breast cancer is performed using mammography, but this diagnostic tool is costly and only feasible in countries with a modern health infrastructure. Clinical breast examination as a low-cost screening alternative is limited and bears several risks, such as not detecting smaller pathologies (often overlooking nascent cancers). Recent studies have identified cancerous findings in samples of otherwise healthy individuals following reduction mammaplasty (RM). RMs are generally electively performed in women with macromastia to reduce the volume of female breasts and in cases following oncologic surgery for breast cancer to adjust the size of the breast(s). In 2018, an estimated number of 534,294 breast reductions were performed worldwide [5]. Although such occult cancer findings in RM specimens [6,7,8,9] have been previously reported, information regarding the incidence of breast cancer found parenthetically in women undergoing breast reduction surgery remains sparse.

The goal of this study was to better determine the incidence of occult breast cancer found during preoperative workup or the pathology assessment of the reduction specimens using a large retrospective cohort of women who underwent surgery within an 11-year period (2000–2010). Such information may allow for the development new screening recommendations. Although the majority of our resected breast tissue specimens were benign, our findings indicate that there is a continued need for histopathological examination. In addition to this, the goal of our study was to outline the importance of developing more feasible screening methods that become commonplace due to this unaddressed risk of cancer development in female patients.

## 2. Methods and Patients

With Institutional Review Board (IRB) approval, we performed an international, multicenter study based on retrospective medical chart review. Twenty-two centers in Germany, Austria, and South-Tyrol (German-speaking part of Italy) were invited to participate in the study. Sixteen hospitals contributed data from 5781 patients who underwent reduction mammaplasty (unilateral or bilateral) mainly because of macromastia. Eligibility was defined as women who had undergone unilateral or bilateral reduction mammaplasty for non-oncologic reasons in the years 2000–2010, and of whom the medical record included, at minimum, the operative report available for review. Women who had undergone unilateral breast reduction to restore symmetry following contralateral breast cancer surgery were included. Women with a history of bilateral breast cancer were excluded.

Baseline characteristics of patients were retrieved from the medical records. Family history of breast/ovarian cancer (BOC) was classified as positive if at least one first-degree relative (FDR) had a diagnosis of breast and/or ovarian cancer. If no information on any of the baseline characteristics or affected relatives was found in the medical record, the respective data point was classified as not available and labeled as missing. Candidates were classified as having undergone preoperative imaging if the radiology report of at least one breast imaging method (mammography, breast ultrasound, breast magnetic resonance imaging) from the preoperative period was available in the medical record. Information on the surgical procedure was attained from the operative report, and information on pathological workup from the pathology report. Pathologic findings in RM specimens were categorized as premalignant (atypical ductal hyperplasia (ADH) or atypical lobular hyperplasia (ALH)), preinvasive (ductal carcinoma in situ (DCIS) or lobular carcinoma in situ (LCIS)), or invasive (ductal carcinoma or lobular carcinoma).

All data were entered remotely at the participating center using an electronic data capture form and stored in a central database. Statistical analysis was mainly descriptive. To test for differences in baseline characteristics between patients with and without preoperative imaging, the *t*-test was used for continuous variables and the chi-squared test for categorical variables. The level of statistical significance was chosen at *p* < 0.05.

## 3. Results

The participating centers identified a total of 5781 eligible patients who underwent reduction mammoplasty between 2000 and 2010. Each center contributed a median number of 323 patients (range: 60–1103 patients). The average age of patients at RM was 38.5 (±13.6) years with a mean body weight of 70.5 (±12.1) kg and a BMI of 25.9 (±4.2) kg/m^2^. A total of 578 patients (~10%) had a history of unilateral breast cancer, of which 450 had been invasive carcinomas and 128 in-situ carcinomas. Family history of breast and/or ovarian cancer in first-degree relatives (FDR) was positive in 112 patients (1.9%). Baseline characteristics of patients are shown in Table 1.

Pre-operative imaging performed with at least one method (mammogram, breast ultrasound, breast MRI) was documented for 1618 patients (28%). Patients who had undergone imaging were on average older (mean age 43.2 years vs. 36.7 years, *p* < 0.001). The proportion of women who had preoperative imaging was higher among postmenopausal vs. premenopausal women (40.2% vs. 24.1%), patients with a personal history of breast cancer (47.9% vs. 25.8% in those with no history), and women who had a family history of BOC (57.1% vs. 33.3%). Patients with imaging had a slightly higher body weight and lower body height, and higher BMI (Table 1). Notably, of the overall 578 patients in our study population who had a history of breast cancer, 301 patients (52.1%) had no preoperative imaging documented. Of the 112 patients with a family history of BOC, 48 patients (42.9%) had no documented imaging preceding the RM. Overall, 4103 patients (71.0%) had preoperative photo documentation of the breasts (Table 2).

**Figure 1:**
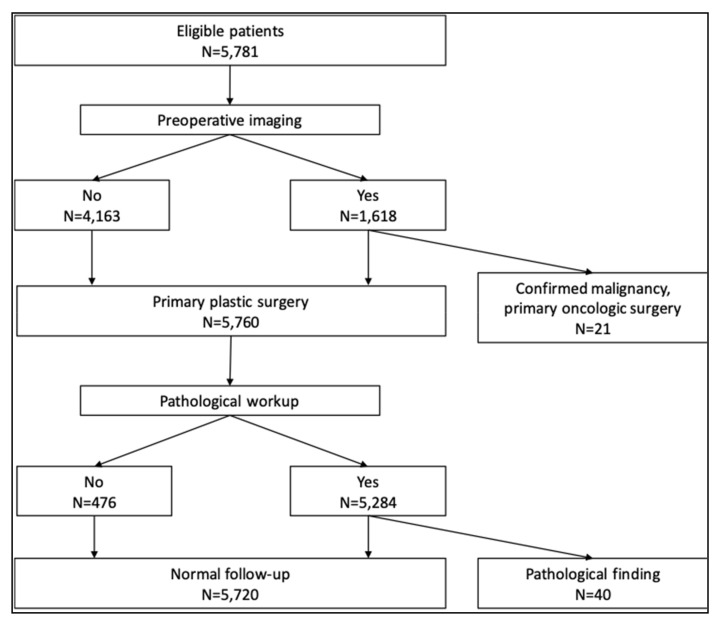
CONSORT diagram of the patient flow through preoperative diagnostic and surgical intervention in the study.

The most frequently used radiological imaging technique was mammography (1438 patients, 24.9%), followed by breast ultrasound (927 patients, 16.0%) and breast MRI (135 patients, 2.3%). Among the 1618 patients who had any preoperative imaging, a radiologically suspect finding was documented for 206 patients (12.7%), and in 21 patients (1.3%) a biopsy-confirmed malignancy was detected. The indication for surgery in these 21 patients became an oncologic indication, therefore they were excluded from further analysis. The operative reports of the remaining 5760 patients were reviewed (Figure 1).

In total, 5027 patients underwent bilateral and 733 patients had unilateral reduction mammoplasties. The most frequent indication for this surgical procedure was macromastia (*n* = 4336 patients, 75.3%; Table 3). Other indications were acquired asymmetry (9.5%), developmental asymmetry (8.5%), breast ptosis (4.2%) and other reasons (3.8%). The applied surgical techniques and their frequencies are listed in Table 3.

A pathological report of the specimen workup was available for 5284 of the 5760 operated patients (91.7%). The specimen weight ranged from 5 g to 6800 g, with a median weight of 496 g. Suture marking was documented in 34 (0.6%) of patients. In 40 of 5284 patients (0.8%), a pathological finding was reported: 13 patients with premalignant lesions, 18 patients with preinvasive cancer, and 9 patients (0.2%) with invasive breast cancer (Table 3).

Of the 13 patients with premalignant histologic findings, 7 were diagnosed with ADH (average age: 49 [±43–62] years) and 6 with ALH (average age: 48.5 [±41–65] years). In this group, 2 patients had been diagnosed with contralateral breast cancer 1 year and 3 years prior to the reduction mammaplasty; none had a positive breast/ovarian cancer family history. Breast imaging prior to the surgery was documented for 3/13 patients (Table 4).

In the group of 18 patients with preinvasive histology findings, 14 were diagnosed with DCIS (average age: 54.5 [±23–65] years) and 4 with LCIS (average age: 57.5 [±44–66] years). Four patients had a history of contralateral breast cancer, and two patients with DCIS had a positive family history of BOC. Seven of 18 patients received a preoperative mamma diagnostic. Seven of 18 patients received a preoperative mamma diagnostic.

There were six patients with ductal (average age: 54.5 [±46–64] years) and three patients with lobular (average age: 50 [±44–53] years) invasive cancer findings. One patient had a history of contralateral breast cancer and one patient a history of contralateral DCIS. Six of the nine patients in whom invasive cancer was detected received a preoperative mamma diagnostic.

Patients with pathological findings in their breast specimens showed an increased median age (51 years) compared to the median age of the total patient population of this study (38 years).

Detailed information on the baseline characteristics of patients with pathological findings can be found in Table 4.

## 4. Discussion

Breast cancer found parenthetically at the time of reduction mammaplasty is uncommon. In this large multicenter study, a pathological finding was detected in 0.8% of patients during workup of the RM specimen, including 0.2% patients with occult invasive breast cancer, confirming a low incidence of breast cancer detected during RM. Older patients were more likely to have a diagnosis of incidentally found breast cancer, though their median age (51 years) was lower than that of the overall breast cancer population (median ~62 years), which is explained by the younger age structure of the study sample [10].

Our study of 5781 women represents a large cohort of patients who underwent reduction mammaplasty. The rate of incidentally found breast cancer was consistent with that reported in prior literature (0.06–4.5%) [7,8,11,12].

Breast imaging prior to surgery was performed in 28% of our patients (*n* = 1618), with 21 patients having been diagnosed with breast cancer by confirmatory biopsies following imaging. In four of the nine patients with occult invasive cancer, the preoperative screening imaging revealed abnormal finding using radiological imaging, but no biopsy was reported, thus the patients underwent plastic surgery (i.e., non-oncologic surgery). Of 27 patients with occult invasive or pre-invasive cancer, only 50% had preoperative screening. Overall, no preoperative screening was documented for the majority of patients (72.0%) undergoing RM. In particular, roughly half of those with a personal or family history of breast cancer did not have preoperative breast screening documented.

In the collective of patients undergoing breast reduction surgery, neoplastic changes of breast tissue are typically not primarily expected. However, breast cancer is the most common malignant cancer in women [2] and the presence of occult breast cancer in RM specimens has been described before [7,8,11,12]. In previous reports of reduction mammoplasty, the presence of a contralateral breast cancer was found to be a major risk factor for the presence of occult carcinoma [12,13,14]. Preoperative radiologic evaluation is an important tool to reduce the number of carcinomas detected by chance during RM. In young patients, the sensitivity of conventional mammography, especially in patients with very dense breasts, is reduced [15,16]. A higher accuracy for breast cancer detection by 3D digital mammography compared to the conventional technique, especially in woman under 50 years of age with dense fibro-glandular tissue could be shown [17]. Adjunctive breast-ultrasound has shown increased screening sensitivity, though false-positives are common [18]. Patients with a known elevated familial risk to develop breast cancer benefit from a significantly higher sensitivity of MRI screening compared to mammography even if combined with ultrasound diagnostics [19]. Campbell et al. analyzed the role of preoperative mammography in women seeking reduction mammoplasty and noted a high incidence of false-positive results, without malignant findings in the specimen [20].

Yet, we believe there is a need for preoperative screening prior to RM, particularly in women with risk factors such as a personal or family history of breast cancer. The choice of screening method would follow the recommendations in established guidelines. Knowledge of and following evidence-based cancer screening guidelines should be mandatory for effective screening and every performing plastic surgeon. Selber et al. found that only two-thirds of breast surgery performing plastic surgeons were familiar with the American Cancer Society Breast Cancer Screening Guidelines, and fewer than half reported concordant practices [21]. Among other recommendations, these guidelines suggest all women at average risk for breast cancer should begin having yearly mammograms by the age of 45 and can change to having mammograms every other year beginning at age 55 [22]. Women should have the choice to start screening with yearly mammograms as early as age 40 if they want to. Further, women who are at high risk for breast cancer based on certain factors should get a breast MRI and a mammogram every year, typically starting at age 30.

In our study, of the overall 578 patients who had a history of breast cancer, 301 patients (52.1%) had no preoperative imaging documented. Of the 112 patients with a family history of breast/ovarian cancer in first-degree relatives, 48 patients (42.9%) had no documented imaging preceding the RM. Taken the American Cancer Society Breast Cancer Screening Guidelines into consideration, we suggest that this number is still too low when performing RM.

Our study benefits from a large sample size of nearly 6000 RM patients that stems from an international, multicenter approach. A significant number of patients had no documented radiological imaging prior to the RM. We assume that some patients had their screening at peripheral institutions and while screening results, presumably negative, may have been known to the surgeons, they were not documented in the medical record and were thus assumed to be not done for the present study. For those patients who had pre-operative imaging, we do not have data on the time of imaging relative to surgery. However, since the study data were collected by reviewing medical records, we make the assumption that if the radiological report was found in the patient’s chart, the imaging was performed close to the date of surgery. The pathological report was not found in 8.3% of the patient medical records that were reviewed. Therefore, the number of patients of whom the pathology report was available was used as the denominator when estimating the incidence of pathologic findings. For several patients, the weight of the surgical sample was only of few grams, possibly such small specimens were intended as representative samples. It is also unknown how much of the breast tissue was histopathologically examined, especially if the reduction sample was large. Both situations pose the risk that some tissue abnormalities went undetected and could lead to an underestimation of the incidence of occult findings, but we believe the difference would be minimal and would not impact the results of our study.

Our study did not compare whether there was a difference in the discovery rate of cancer according to the method of RM. Rietjens et al. did not find any difference in the discovery rate between superior or inferior pedicle mammaplasty [23]. Unfortunately, we do not have follow-up date of patients to determine the incidence of post-RM breast cancer and relate this to the preoperative screening and completeness of pathological workup.

In conclusion, a large proportion of RM patients are not screened before undergoing RM and undetected breast pathology is found, albeit with low incidence, in RM specimens. Preoperative imaging should be routinely performed in patients scheduled for RM, especially those with risk factors of breast cancer, to identify and enable a primary oncologic approach.

## Figures and Tables

**Table 1:** Baseline characteristics of patients (*n* = 5781).

		Preoperative Imaging		
Characteristic	Total	Yes	No	*p*-Value *
Age at RM ^1^, *n* (%)	5781 (100)	1618 (28.0)	4163 (72.0)	
Median (range), years	38 (14–79)	43 (15–79)	36 (14–78)	
Mean (SD), years	38.5 (13.6)	43.2 (12.8)	36.7 (13.4)	<0.001
Body weight, *n* (%)	3741 (64.7)	1268 (33.9)	2473 (66.1)	
Median (range), kg	69 (41–165)	70 (45–158)	69 (41–165)	
Mean (SD), kg	70.5 (12.1)	71.4 (12.3)	71.4 (12.3)	0.003
N/A	2040 (35.3)	–	–	
Body height, *n* (%)	3712 (64.2)	1254 (33.8)	2458 (66.2)	
Median (range), cm	165 (133–197)	165 (145–197)	165 (133–195)	
Mean (SD), cm	164.9 (6.5)	164.7 (6.6)	165.0 (6.5)	0.005
N/A	2068 (35.8)	–	–	
BMI (kg/m^2^), *n* (%)	3692 (63.9)	1243 (33.7)	2449 (66.3)	
Median (range), kg/m^2^	25 (14–58)	27 (16–52)	25 (14–58)	
Mean (SD), kg/m^2^	25.9 (4.2)	26.3 (4.3)	25.7 (4.2)	<0.001
N/A	2089 (36.1)	–	–	
Menopausal status, *n* (%)				
Premenopausal	3295 (57.0)	795 (24.1)	2500 (75.9)	
Postmenopausal	810 (14.0)	326 (40.2)	484 (59.8)	<0.001
N/A	1676 (29.0)			
Personal history of breast cancer, *n* (%)				
No	5203 (90.0)	1341 (25.8)	3862 (74.2)	
Yes	578 (10.0)	277 (47.9)	301 (52.1)	<0.001
in situ	128 (2.2)	54 (42.2)	74 (57.8)	
Invasive	450 (7.8)	223 (49.6)	227 (50.4)	
Family history of breast and/or ovarian cancer in FDRs ^2^, *n* (%)				
Positive	112 (1.9)	64 (57.1)	48 (42.9)	
Negative	2096 (36.3)	697 (33.3)	1399 (66.7)	<0.001
N/A	3573 (61.8)	–	–	

Abbreviations: RM, reduction mammoplasty; SD, standard deviation; N/A, not available; BMI, body mass index; FDR, first-degree relative. ^1^ Age calculated as year of surgery minus birth year; ^2^ First-degree relatives are mother or sister. No cases of BC were reported in the father, brother, daughter, or son of any patient undergoing RM; * *p*-value for difference between patients with and without imaging (*t*-test for continuous, chi^2^ for categorical variables).

**Table 2:** Preoperative imaging (*n* = 5781).

Radiologic Procedure *	*n*	%
Mammogram	1438	24.9
Breast ultrasound	927	16.0
Breast MRI	135	2.3
Any preoperative imaging	1618	28.0
Suspect finding	206	12.7
Biopsy positive	21	1.3
**Photo documentation**		
Yes	4103	71.0
No	1678	29.0

Abbreviation: MRI, magnetic resonance imaging; * Patients who had more than one radiologic procedure are counted in each category.

**Table 3:** Details of surgical procedure and pathological workup (*n* = 5760).

	*n*	%
Reduction mammoplasty		
Bilateral	5027	87.3
Unilateral	733	12.7
Indication ^1^		
Macromastia	4336	75.3
Acquired asymmetry	545	9.5
Developmental asymmetry	490	8.5
Breast ptosis	241	4.2
Other	219	3.8
Surgical technique ^2^		
Superior pedicle techniques	2762	48.0
Inferior pedicle techniques	1345	23.4
Vertical scar technique	665	11.5
Central pedicle technique	296	5.1
Medio cranial pedicle techniques	241	4.2
Short scar technique	143	2.5
Free nipple	93	1.6
Round block	83	1.4
Reconstructive interventions (implants, corrections)	60	1.0
Other or insufficiently described techniques	161	2.8
Pathological analysis documented		
Yes	5284	91.7
No	476	8.3
Orientation marking of specimen		
Yes	34	0.6
No	5726	99.4
Pathological finding		
Yes	40	0.8
Premalignant	13	0.3
Preinvasive	18	0.3
Invasive Cancer	9	0.2
No	5244	99.2
Weight of specimen	*n* = 9415 *	87.3
Median (range), g	496 (5–6800)	
Mean (SD), g	548 (342)	

Abbreviation: SD, standard deviation; ^1^ In 71 patients undergoing bilateral RM, the indication was different for each breast. These patients were counted in each category; ^2^ In 89 patients undergoing bilateral RM, a different technique was applied on each breast. These patients were counted in each category; * From a total of 10,787 operated breasts (5027 patients had bilateral RM and 733 patients had unilateral RM), weight of tissue specimen was available of 9415 breasts.

**Table 4:** Baseline characteristics of patients with pathological findings.

Histology	*n*	Median Age (Range)	History of Cancer *n* (Years Before RM) ^1^	Family History ^2^	Indication	Preoperative Imaging *n* (Method and Result)^3^	Year of RM (Range)
Premalignant	13						
ADH	7 *	49 (43–62)	BC: 1 (1 Year)	Pos: 0 Neg: 1 N/A: 6	Acquired asymmetry: 1 Macromastia: 6	Yes: 3 (1 pt: MG+, US+, MRI−; 1 pt: MG−; 1 pt: US−) No: 4	2000–2010
ALH	6	48.5 (41–65)	BC: 1 (3 Years)	Pos: 0 Neg: 1 N/A: 5	Acquired asymmetry: 1 Macromastia: 5	Yes: 0 No: 6	2001- 2010
Preinvasive	18						
DCIS	14 *	54.5 (23–65)	BC: 3 (2, 3, 18 Years)	Pos: 2 Neg: 3 N/A: 9	Acquired asymmetry: 3 Developmental asymmetry: 1 Macromastia: 10	Yes: 6 (5 pts: MG−, 1 pt: US−) No: 8	2004–2010
LCIS	4 *	57.5 (44–66)	BC: 1 (2 Years)	Pos: 0 Neg: 2 N/A: 2	Acquired asymmetry: 1 Macromastia: 3	Yes: 1 (MG−, US−, MRI−) No: 3	2004–2007
Invasive	9						
Ductal	6	54.5 (46–64)	BC: 1 (5 Years)	Pos: 1 Neg: 0 N/A: 5	Acquired asymmetry: 1 Macromastia: 5	Yes: 5 (1 pt: MG+, US+; 2 pts: MG+; 2 pts: MG−, US−) No: 1	2000–2010
Lobular	3	50 (44–53)	in situ: 1 (1 Year)	Pos: 0 Neg: 0 N/A: 3	Acquired asymmetry: 2 Macromastia: 1	Yes: 1 (US+, MRI−) No: 2	2000–2004

Abbreviations: ADH, atypical ductal hyperplasia; ALH, atypical lobular hyperplasia; DCIS, ductal carcinoma in situ; LCIS, lobular carcinoma in situ; BC, breast cancer; MG, mammogram; US, breast ultrasound; MRI, breast magnetic resonance imaging; RM, reduction mammoplasty; N/A, not available; ^1^ Patients with a history of contralateral cancer were allowed in the study; ^2^ Family history was considered positive if the patient had at least one first-degree-relative affected with breast and/or ovarian cancer; ^3^ Listed are exams of which a radiology report was available in the patient medical record. A ‘+’ denotes a suspect finding, a “−” denotes a negative result in the written report; * In 6 patients the pathology was found bilaterally: ADH (2 patients), DCIS (3 patients), LCIS (1 patient).
